# Review no. 3: handling of longitudinal creatinine data to define acute kidney injury

**DOI:** 10.1007/s10157-026-02856-3

**Published:** 2026-04-22

**Authors:** Yoshihisa Miyamoto, Yuka Sugawara, Megumi Oshima, Hajime Nagasu, Takashige Kuwabara, Tadashi Sofue, Naoki Nakagawa, Masao Iwagami

**Affiliations:** 1 Department of Real-World Evidence, The University of Tokyo, Tokyo, Japan; 2 Division of Nephrology and Endocrinology, The University of Tokyo, Tokyo, Japan; 3 Subcommittee for the Promotion of Clinical Research, The Japanese Society of Nephrology, Tokyo, Japan; 4 Department of Nephrology and Rheumatology, Kanazawa University, Kanazawa, Japan; 5 Department of Nephrology and Hypertension, Kawasaki Medical School, Okayama, Japan; 6 Department of Nephrology, Kumamoto University Graduate School of Medical Sciences, Kumamoto, Japan; 7 Department of Cardiorenal and Cerebrovascular Medicine, Faculty of Medicine, Kagawa University, Kagawa, Japan; 8 Division of Cardiology and Nephrology, Department of Internal Medicine, Asahikawa Medical University, Asahikawa, Japan; 9 Department of Digital Health, Institute of Medicine, University of Tsukuba, Ibaraki, Japan; 10 Department of Non-Communicable Disease Epidemiology, Faculty of Epidemiology and Population Health, London School of Hygiene and Tropical Medicine, London, UK

**Keywords:** Acute kidney injury, Serum creatinine, Real-world data, KDIGO criteria

## Abstract

Acute kidney injury (AKI) is characterized by a sudden decline in kidney function. The Kidney Disease: Improving Global Outcomes (KDIGO) clinical practice guidelines define AKI based on an increase in serum creatinine (SCr) levels as well as a decrease in urine volume. For clinical researchers of AKI, the ability to handle longitudinal SCr data and flag AKI status and stage in individual patients is a fundamental skill. This article provides a practical guide for identifying AKI episodes from longitudinal SCr data using R programming. The methods described in this article are based on a hands-on seminar presented at the 68th Annual Meeting of the Japanese Society of Nephrology in 2025.

**Supplementary Information:**

The online version contains supplementary material available at 10.1007/s10157-026-02856-3.

## Introduction

Acute kidney injury (AKI) is a clinical syndrome characterized by a rapid decrease in kidney function occurring over hours to days [1]. Following the Risk, Injury, Failure, Loss, End-Stage Renal Disease (RIFLE) criteria, the Acute Kidney Injury Network (AKIN) criteria, and the Kidney Disease: Improving Global Outcomes (KDIGO), the Acute Kidney Injury Work Group published its Clinical Practice Guideline for AKI in 2012, and these criteria have been widely recognized and used for clinical research and practice [2]. The KDIGO defines AKI as any of the following: an increase in serum creatinine (SCr) by ≥ 0.3 mg/dL within 48 h, an increase in SCr to ≥ 1.5 times baseline, which is known or presumed to have occurred within the prior 7 days, or a urine volume < 0.5 mL/kg/h for 6 h. Although both SCr and urine volume are included in the definition of AKI, many researchers primarily use SCr because of the practical challenges of reliably and constantly collecting urine output data [3].

In recent randomized clinical trials (RCTs), the KDIGO AKI criteria have been frequently used for patient enrollment or as an endpoint. For instance, AKI stages 2 or 3 served as eligibility for enrollment in RCTs evaluating strategies for initiating kidney replacement therapy, such as the AKIKI [4], ELAIN [5], IDEAL-ICU [6], and STAART-AKI trials [7]. AKI has also been regarded as a primary endpoint in trials evaluating preventive treatments, such as the PROTECTION trial [8]. Likewise, in the CONFIRM trial evaluating terlipressin for hepatorenal syndrome (HRS)-associated AKI [9], the reversal of HRS-associated AKI—defined as two consecutive SCr measurements ≤ 1.5 mg/dL—serves as an endpoint indicating improvement.

In observational studies, researchers have described the incidence of AKI [10], examined the risk factors for AKI [11], or predicted the incidence of AKI [12]. Some researchers regard AKI as an exposure of interest for examining short- and long-term consequences after AKI, such as a composite endpoint of death, new kidney replacement therapy, or persistent renal dysfunction [13].

Handling longitudinal SCr data is a fundamental skill for clinical researchers studying AKI in various settings. Applying the KDIGO AKI SCr criteria is straightforward, particularly when a unique baseline SCr level is readily determined. For example, when identifying hospital-acquired AKI (HA-AKI) after elective surgery, SCr level measurement immediately before surgery is an appropriate baseline. Meanwhile, determining AKI in cases of unplanned admission for acute illnesses, such as sepsis and volume depletion, often necessitates the comparison of admission SCr with an outpatient SCr. In addition, the original KDIGO criteria use an increase in SCr to ≥ 1.5 times baseline “within the prior 7 days.” However, SCr measurement within the previous 7 days may not always be available, especially in unplanned or emergency admissions, as well as in outpatient or emergency room settings, where community-acquired AKI (CA-AKI) is common [14]. Accordingly, some researchers have developed methods to define baseline SCr more flexibly, such as using the most recent outpatient SCr or the mean or median of SCr measurements from a window of 7–365 days before the first inpatient measurement [15, 16].

This tutorial paper aims to equip readers with the basic skills to handle longitudinal SCr data to identify AKI episodes by flagging AKI status and stage using the statistical software R. The intended readership of this manuscript includes clinical researchers, nephrology trainees, and investigators working with longitudinal serum creatinine data. We provide a hypothetical dataset of 1000 patients (Appendix 1) and example codes (scripts) in R (version 4.3.3; R Foundation for Statistical Computing) (Appendix 2). The methods described in this article are based on a hands-on seminar presented at the 68th Annual Meeting of the Japanese Society of Nephrology in 2025 [17].

### Sample dataset

The hypothetical dataset comprises virtual outpatient and inpatient SCr values for 1000 individuals from a hospital (Appendix 1). This dataset is structured in a long format in which each patient’s multiple SCr measurements at different times are recorded in separate rows. When loaded into R, this dataset (referred to as df) contains 11,696 rows and 4 columns. The columns include.

- **id**: The patient identification number. Each patient is assigned a unique ID that allows for the identification of a specific patient across multiple measurements. The dataset contains 1000 unique patient IDs.
- **date**: The measurement date, in “YYYY-MM-DD” format.
- **creatinine:** the SCr value for each measurement.
- **inpatient:** a flag indicating the patient’s status at the time of measurement. A value of 0 signifies an outpatient measurement and 1 indicates an inpatient measurement. In this sample dataset, we assume that the first inpatient creatinine level was measured upon admission.

For simplicity, the provided hypothetical dataset includes only one measurement per day. In a real-world dataset, some patients may have undergone two or more measurements per day. Researchers would appropriately order these measurements and select the earliest inpatient SCr measurement, particularly on the day of admission, if additional information (e.g., the time of day) is available.

### Objectives and workflow to identify AKI from longitudinal SCr data

Generally, there are two approaches for handling baseline SCr levels to define AKI: the static baseline approach and the dynamic baseline approach [18–20]. The static baseline approach uses a fixed reference SCr value (at a single time point) obtained before hospitalization, surgery, or an acute illness episode. In contrast, the dynamic baseline approach applies a rolling time window that continuously updates the baseline reference when new SCr measurements become available, particularly during hospitalization.

**Fig. 1:**
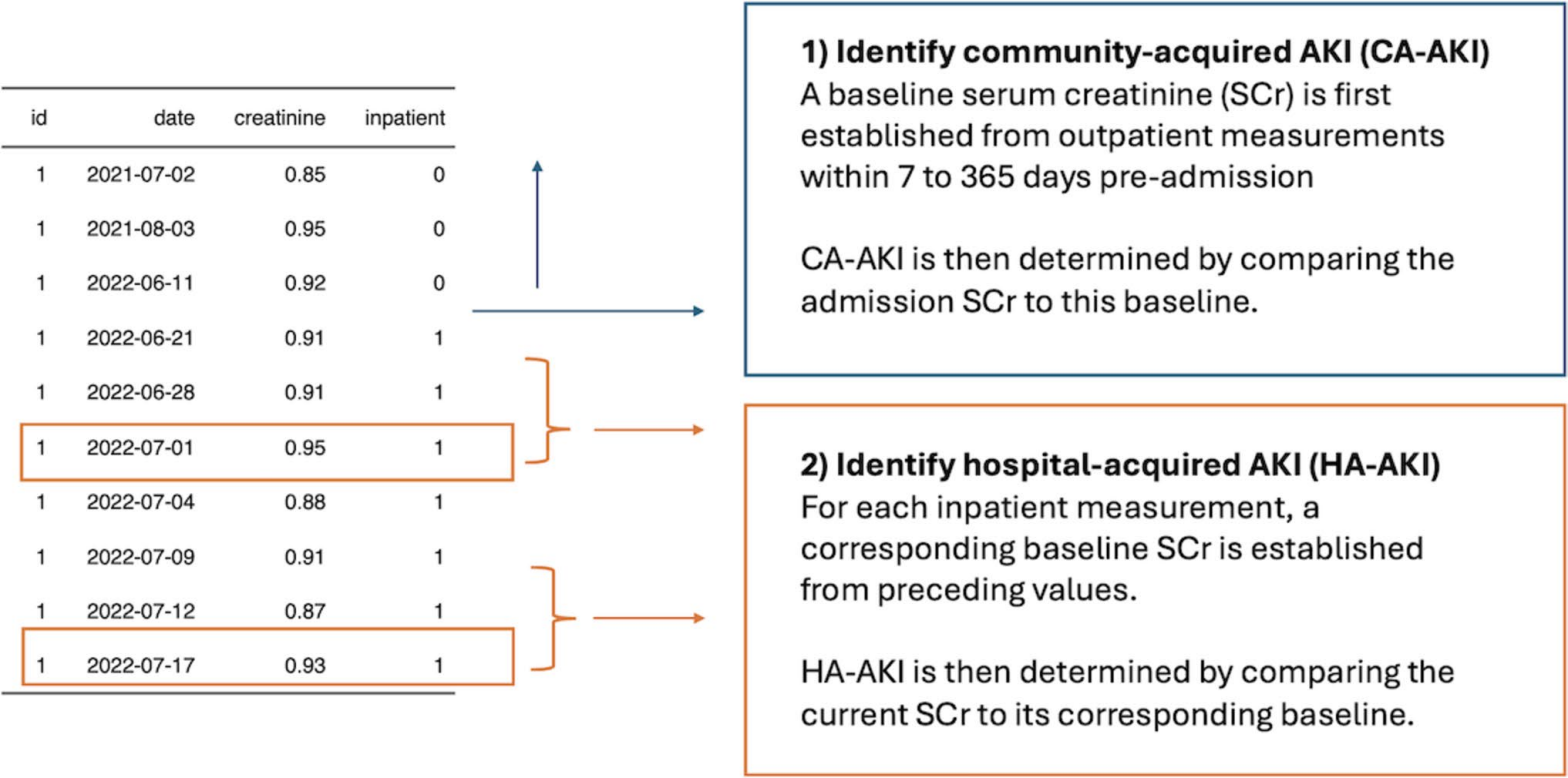
The visual abstract of the workflow in this tutorial

As shown in Fig. 1, the goal of this tutorial is to identify (i) CA-AKI at admission based on the static baseline approach and (ii) HA-AKI using the dynamic baseline approach. More specifically, for CA-AKI, we determine the AKI status and stage at admission using their outpatient SCr values. The baseline SCr level is defined as the most recent outpatient SCr level measured during the period from 7 to 365 days before admission. In contrast, to flag the presence or absence of HA-AKI, we compare each inpatient SCr measurement to the minimum SCr value observed in the past 7 days (for a ≥ 1.5-fold increase) or the minimum SCr value in the past 2 days (for a ≥ 0.3 mg/dL increase). Similarly, we define AKI stages by comparing the SCr values at each measurement to the minimum SCr value observed within the preceding 7 days. The patient’s overall AKI stage during hospitalization is considered the maximal stage.

Although this tutorial focuses on a specific methodology, other approaches may also be valid. For instance, using a fixed static baseline to assess HA-AKI can be justified depending on the research context (e.g., in the assessment of cardiovascular surgery-associated AKI after elective surgery) and data availability.

In this tutorial, we use the *tidyverse* package (version 2.0.0) for data manipulation and visualization and the *slider* package (version 0.3.2) to implement the rolling time window to define a baseline for each inpatient SCr measurement by looking back over the preceding 48 h or 7 days.

### Identification of community-acquired AKI (CA-AKI)

**1. Identifying the first inpatient measurement and date**: first, we determine the date of the earliest (first) inpatient SCr measurement from the longitudinal dataset. This date is stored as the first_inp_date variable. It is assigned across all the rows belonging to that individual to ensure a consistent reference point.

**2. Calculating the number of days from the first inpatient date**: a new column, diff_inp, is created to indicate the number of days between each date of SCr measurement and the first_inp_date. Negative values indicate that the measurements were taken before admission.

**3. Flagging outpatient SCr measurements during the baseline window**: a flag, baseout_flag, is created to identify outpatient SCr measurements taken within a specific window (from 7 to 365 days in the present tutorial, which can be flexibly changed by researchers) before the first_inp_date (Table 1).

**Table 1:** Data structure after identifying the date of the first inpatient serum creatinine measurement

id	date	creatinine	inpatient	first_inp_date	diff_inp	baseout_flag
1	2021–07-02	0.85	0	2022–06–21	− 354	1
1	2021–08-03	0.95	0	2022–06–21	− 322	1
1	2022–06–11	0.92	0	2022–06–21	− 10	1
1	2022–06–21	0.91	1	2022–06–21	0	0
1	2022–06–28	0.91	1	2022–06–21	7	0
1	2022–07-01	0.95	1	2022–06–21	10	0
1	2022–07-04	0.88	1	2022–06–21	13	0
1	2022–07–09	0.91	1	2022–06–21	18	0
1	2022–07–12	0.87	1	2022–06–21	21	0
1	2022–07–17	0.93	1	2022–06–21	26	0

*id* Unique patient identification number, *date* Date of serum creatinine measurement, *creatinine* Serum creatinine concentration (mg/dL), *inpatient* Binary indicator for hospitalization status (1 = inpatient, 0 = outpatient), *first*_*inp*_*date* The date of the patient’s first recorded inpatient serum creatinine measurement, *diff*_*inp* Number of days from the first inpatient measurement date, *baseout_flag* Binary indicator for the availability of outpatient measurements within the baseline window (7 to 365 days prior to the first inpatient date)

**4. Determining the baseline SCr**: if there are ≥1 measurements within the baseline window (baseout_flag = 1 in Table 1), the SCr value from the measurement closest to the first_inp_date is used as the baseline SCr (baseline_out_creat). If no such outpatient data are available, the first SCr value recorded during hospitalization is used. The timing (number of days from the first inpatient measurement date) of the baseline SCr measurement is also determined (max_date_base). This process consolidates each patient’s data into a single row within a new data frame, df_base (Table 2). Each row contains the unique baseline SCr (baseline_out_creat) and the corresponding measurement date (max_date_base) for each individual.

**Table 2:** The most recent outpatient serum creatinine measurement from 7 to 365 days before the date of the first inpatient serum creatinine measurement

id	max_date_base	baseline_out_creat
1	− 10	0.92
2	− 92	1.10
3	− 195	2.36
4	− 27	1.29
5	− 77	1.34
6	− 70	1.48
7	− 311	0.57
8	− 15	0.85
9	− 56	1.02
10	− 38	1.96

*id* Unique patient identification number, *max*_*date*_*base* The timing (number of days from the first inpatient measurement date) on which the baseline_out_creat was recorded, *baseline*_*out*_*creat* The outpatient serum creatinine value measured closest to the admission date within the 7–365-day window, used as the static baseline for community-acquired AKI

**5. Merging the baseline SCr with the original dataset**: to compare the first inpatient SCr value (creatinine) in the original data frame (df) to the baseline SCr value (baseline_out_creat) in df_base, df_base is added (“left-joined”) to df. Because the original dataset is in a long format, this merge populates the same baseline value for each patient across all corresponding rows (Table 3).

**Table 3:** Data structure after merging the longitudinal inpatient dataset and the baseline (outpatient) serum creatinine dataset

id	date	creatinine	inpatient	first_inp_date	first_inp_flag	diff_inp	baseout_flag	max_date_base	baseline_out_creat
1	2021–07-02	0.85	0	2022–06–21	0	− 354	1	− 10	0.92
1	2021–08-03	0.95	0	2022–06–21	0	− 322	1	− 10	0.92
1	2022–06–11	0.92	0	2022–06–21	0	− 10	1	− 10	0.92
1	2022–06–21	0.91	1	2022–06–21	1	0	0	− 10	0.92
1	2022–06–28	0.91	1	2022–06–21	0	7	0	− 10	0.92
1	2022–07-01	0.95	1	2022–06–21	0	10	0	− 10	0.92
1	2022–07-04	0.88	1	2022–06–21	0	13	0	− 10	0.92
1	2022–07–09	0.91	1	2022–06–21	0	18	0	− 10	0.92
1	2022–07–12	0.87	1	2022–06–21	0	21	0	− 10	0.92
1	2022–07–17	0.93	1	2022–06–21	0	26	0	− 10	0.92

*id* unique patient identification number, *date* date of serum creatinine measurement, *creatinine* serum creatinine concentration (mg/dL), *inpatient* binary indicator for hospitalization status, *first*_*inp*_*date* the date of the patient’s first recorded inpatient serum creatinine measurement, *first*_*inp*_*flag* binary indicator identifying the record of the first inpatient measurement, *diff*_*inp* number of days from the first inpatient measurement date, *baseout*_*flag* binary indicator for outpatient measurements within the baseline window, *max*_*date*_*base* timing (number of days from the first inpatient measurement date) of the selected baseline measurement, *baseline*_*out*_*creat* assigned static baseline serum creatinine value for the patient

**6. Identifying the CA-AKI status**: We first check for the presence or absence of AKI at the first inpatient measurement. A new data frame (Table 4), df_inp, is created by filtering the merged data frame to include only inpatient records (inpatient = 1 in Table 3). This new data frame (df_inp) is grouped by id, and each patient’s records are sorted chronologically by date. A new column, order, is added to number sequential inpatient measurements for each patient.

**Table 4:** Data structure to identify and classify the stage of community-acquired and hospital-acquired AKI

id	diff_inp	creatinine	baseline_out_creat	order	aki_base	aki_s1_ca	aki_s2_ca	aki_s3_ca	min_7d	min_2d	aki_s2_ha	aki_s3_ha	aki_s1_ha
10	0	2.03	1.96	1	0	0	0	0	Inf	Inf	0	0	0
10	6	2.08	1.96	2	0	0	0	0	2.03	Inf	0	0	0
10	7	4.57	1.96	3	0	0	0	0	2.03	2.08	1	1	1
10	8	3.34	1.96	4	0	0	0	0	2.08	2.08	0	0	1
10	11	2.12	1.96	5	0	0	0	0	2.08	Inf	0	0	0
10	15	2.09	1.96	6	0	0	0	0	2.12	Inf	0	0	0
10	18	2.05	1.96	7	0	0	0	0	2.09	Inf	0	0	0
10	21	2.08	1.96	8	0	0	0	0	2.05	Inf	0	0	0
10	25	2.03	1.96	9	0	0	0	0	2.05	Inf	0	0	0
10	26	2.10	1.96	10	0	0	0	0	2.03	2.03	0	0	0
10	28	2.11	1.96	11	0	0	0	0	2.03	2.10	0	0	0

When a patient has no measurements within the look-back window (the past 2 or 7 days), the *slider* package passes an empty vector (length 0) to your function. The code is explicitly instructed to return + Inf (e.g. infinity) whenever this period is empty because the code specifically defines ifelse(length(*x*) = = 0, Inf, …)

*id* Unique patient identification number, *diff*_*inp* number of days from the first inpatient measurement date, *creatinine* serum creatinine concentration (mg/dL), *baseline*_*out*_*creat* The static baseline serum creatinine value, *aki*_*base* binary indicator for community-acquired AKI (1 = creatinine ≥ 1.5 × baseline_out_creat on the first inpatient day), *aki*_*s1*_*ca* binary indicator for community-acquired AKI stage 1, *aki*_*s2*_*ca* binary indicator for community-acquired AKI stage 2, *aki*_*s3*_*ca* binary indicator for community-acquired AKI stage 3, *min*_*7d* rolling minimum serum creatinine value within the previous 7 days (dynamic baseline), *min*_*2d* rolling minimum serum creatinine value within the preceding 2 days (dynamic baseline), *aki*_*s1*_*ha* binary indicator for hospital-acquired AKI stage 1, *aki*_*s2*_*ha* binary indicator for hospital-acquired AKI stage 2, *aki*_*s3*_*ha* binary indicator for hospital-acquired AKI stage 3

A new binary column named aki_base is added. A value of 1 is assigned if it is the patient’s first inpatient SCr measurement (order = 1) and the SCr value is 1.5 times or larger than their baseline SCr value (creatinine ≥ 1.5 * baseline_out_creat). Otherwise, a value of zero is assigned.

**7. Identifying the CA-AKI stage:** Specific flags are also calculated: aki_s1_ca, aki_s2_ca, and aki_s3_ca, corresponding to AKI Stages 1, 2, and 3, respectively. These stage-specific flags are set to 1 only if CA-AKI was flagged above (aki_base = 1) and the earliest (first) inpatient SCr level at admission met the threshold for that stage relative to the baseline SCr value (baseline_out_creat). Specifically, as shown in Table 4,

- AKI stage 1: aki_s1_ca is 1 if creatinine ≥ 1.5 times baseline_out_creat and aki_base is 1.
- AKI stage 2: aki_s2_ca is 1 if creatinine ≥ 2 times baseline_out_creat and aki_base is 1.
- AKI stage 3: aki_s3_ca is 1 if creatinine ≥ 3 times baseline_out_creat or ≥ 4.0 mg/dL, and aki_base is 1.

Notably, in step 4 (“determining the baseline SCr”), we introduced the simplest method to define the baseline SCr. For patients without outpatient data from 7 to 365 days, the baseline SCr level was the first SCr value recorded during hospitalization. Meanwhile, the KDIGO AKI guideline suggests assuming that these patients had an estimated glomerular filtration rate of 75 mL/min/1.73 m^2^ [2]. However, this method may overestimate the frequency of HA-AKI because some patients (especially older patients) without outpatient data may have a glomerular filtration rate lower than 60 mL/min/1.73 m^2^. Some researchers have assumed that baseline SCr values correspond to the lowest SCr values during hospitalization [21]. Because there are no established methods to define baseline SCr when the measured SCr is not available before episodes of interest, researchers may be advised to conduct sensitivity analyses testing two or more methods to determine whether (to what extent) their study results and conclusions change depending on each assumption [10].

### Identification of hospital-acquired AKI (HA-AKI)

**1. Calculating the rolling minimum SCr values**: for HA-AKI, the script uses the existing *slider* package to calculate two rolling minimum SCr values for each SCr measurement during hospitalization: the minimum value over the preceding 7 days (min_7d) and the minimum value over the preceding 2 days (min_2d).

**2. Applying the AKI criteria**: this assessment is performed for every subsequent measurement obtained during the patient’s stay. Instead of a static outpatient baseline, it uses dynamic rolling minimum as reference points to detect new AKI episodes. The script creates flags for different AKI stages based on these specific criteria. Specifically, as shown in Table 4,

- AKI stage 1: aki_s1_ha is 1 if creatinine ≥ 1.5 times the min_7d or an absolute increase of ≥ 0.3 mg/dL compared to the 2-day rolling minimum (min_2d).
- AKI stage 2: aki_s2_ha is 1 if creatinine ≥ 2 times min_7d.
- AKI stage 3: aki_s3_ha is 1 if creatinine ≥ 3 times min_7d or ≥ 4.0 mg/dL.

### Visualization and summarization of the results

**Fig. 2:**
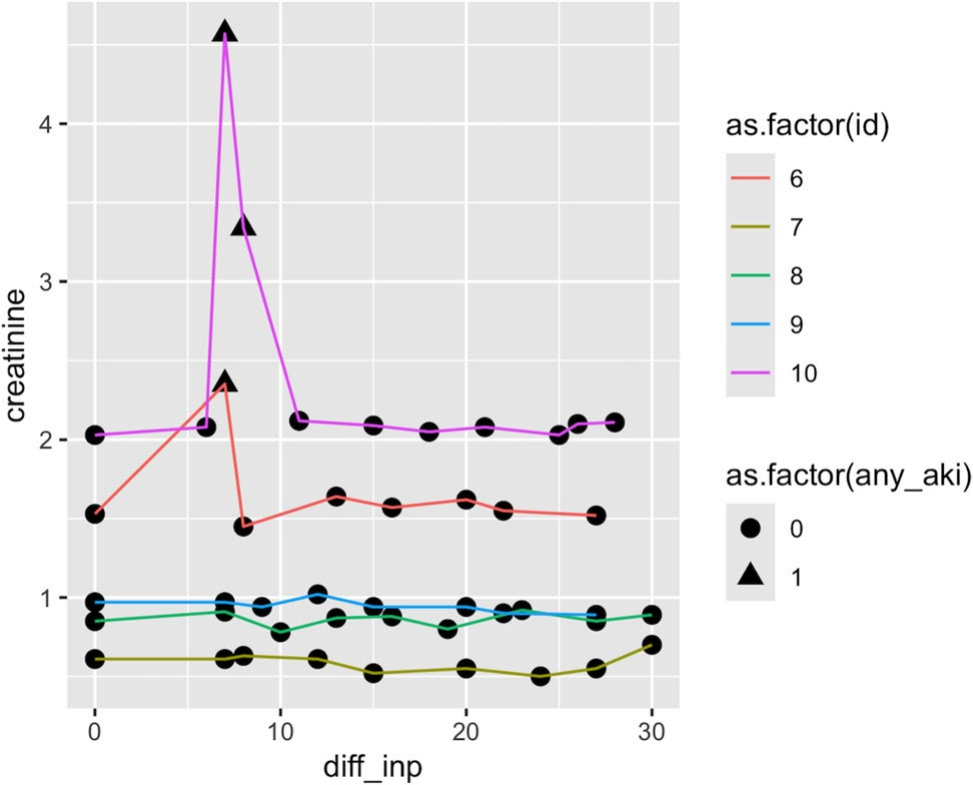
An example of serum creatinine trajectories

In the previous sections, we identified AKI episodes and added the corresponding flags to the inpatient data frame (df_inp) (Table 4). By plotting the SCr values (creatinine) according to the days since the first date of inpatient measurement (diff_inp), we can visualize the trajectories of the selected patients (Fig. 2). Including the flag of the AKI status (any_aki) helps us confirm that our flagging process worked correctly.

Because the dataset is in a long format with multiple rows for each patient, the next step is to aggregate this information. The provided R codes in Appendix 2 create a new summary data frame called df_ind with a single row for each unique patient ID (id). Then, for each patient, the summarise() function in R calculates the maximum value for various AKI-related flag columns (e.g., any_aki, aki_s1_ha, aki_s1_ca). Using the max() function on these binary (0 or 1) flags effectively determines whether a patient has experienced any form of AKI or specific types and stages of CA-AKI or HA-AKI across all recorded measurements. The resulting data frame is then sorted according to patient id (Table 5).

**Table 5:** Data structure to summarize all identified (community-acquired and hospital-acquired) AKI episodes for individual patients

id	any_aki	aki_base	aki_s1_ha	aki_s2_ha	aki_s3_ha	aki_s1_ca	aki_s2_ca	aki_s3_ca	aki_max_stage_ha	aki_max_stage_ca
1	0	0	0	0	0	0	0	0	0	0
2	0	0	0	0	0	0	0	0	0	0
3	0	0	0	0	0	0	0	0	0	0
4	0	0	0	0	0	0	0	0	0	0
5	1	1	0	0	0	1	0	0	0	1
6	1	0	1	0	0	0	0	0	1	0
7	0	0	0	0	0	0	0	0	0	0
8	0	0	0	0	0	0	0	0	0	0
9	0	0	0	0	0	0	0	0	0	0
10	1	0	1	1	1	0	0	0	3	0

*id* Unique patient identification number, *any*_*aki* binary indicator for any acute kidney injury during the observation period, *aki*_*base* binary indicator for community-acquired AKI (1 = creatinine ≥ 1.5 × baseline_out_creat on the first inpatient day), *aki*_*s1*_*ha* binary indicator for hospital-acquired AKI stage 1, *aki*_*s2*_*ha* binary indicator for hospital-acquired AKI stage 2, *aki*_*s3*_*ha* binary indicator for hospital-acquired AKI stage 3, *aki*_*s1*_*ca* binary indicator for community-acquired AKI stage 1, *aki*_*s2*_*ca* binary indicator for community-acquired AKI stage 2, *aki*_*s3*_*ca* binary indicator for community-acquired AKI stage 3, *aki*_*max*_*stage*_*ha* the maximum stage (0–3) of hospital-acquired AKI reached during the patient’s stay, *aki*_*max*_*stage*_*ca* the maximum stage (0–3) of community-acquired AKI identified at the time of admission

To identify the maximum stage of AKI for each patient, two new columns are created: aki_max_stage_ha and aki_max_stage_ca. This works by identifying the most severe stage of AKI. More specifically, a patient is assigned “3” if they meet the criteria for AKI stage 3, even if they also qualify for lower stages. If they do not have AKI stage 3, the code checks for AKI stage 2 and then stage 1. If they do not have AKI stage 3 or 2, the code then checks for AKI stage 1. If a patient shows no flags of AKI stages 1 to 3, they are assigned “0”. This process is performed separately for both HA-AKI and CA-AKI data to determine the peak AKI stage in each context.

**Fig. 3:**
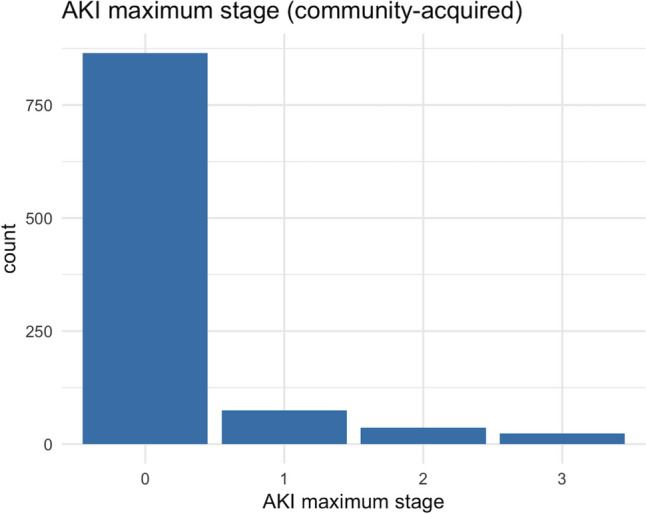
The distribution of the stages of community-acquired AKI in the hypothetical dataset

**Fig. 4:**
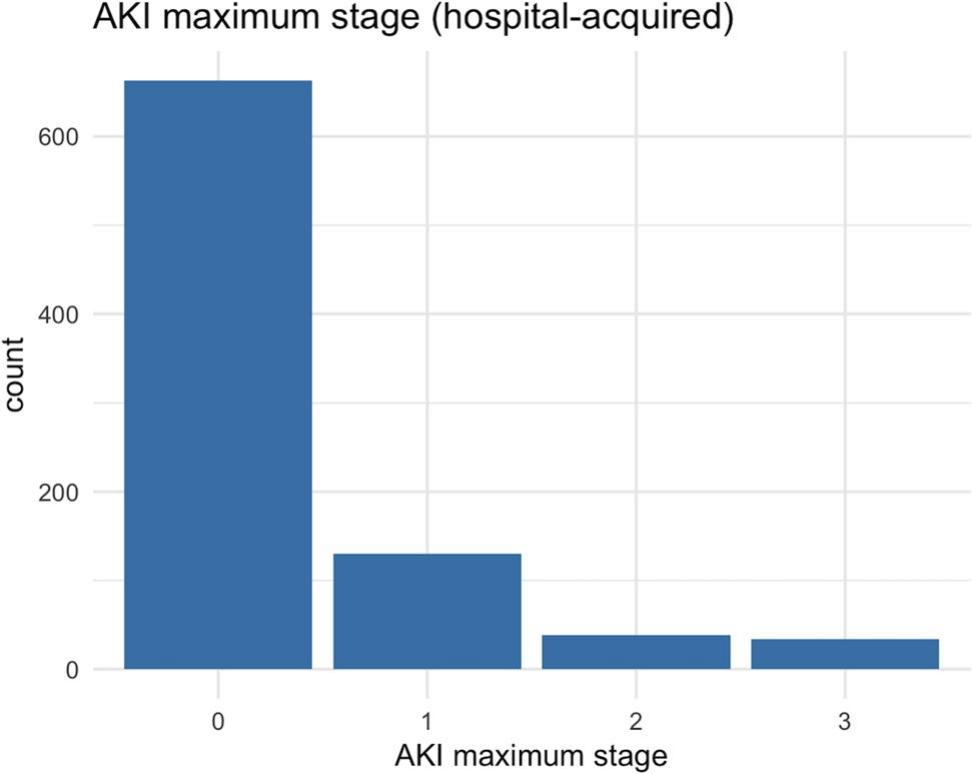
The distribution of the maximum stage of hospital-acquired AKI in the hypothetical dataset

In the hypothetical dataset, among the 1000 patients, 865 had no CA-AKI, 75 had AKI stage 1, 36 had stage 2, and 24 had stage 3 (Fig. 3). Among the 865 patients without CA-AKI, 663 did not develop HA-AKI, 130 reached AKI stage 1, 38 reached AKI stage 2, and 34 reached AKI stage 3 (Fig. 4).

## Conclusions

In this tutorial, we presented a practical and reproducible workflow for identifying and staging CA-AKI and HA-AKI from longitudinal SCr data using R. By leveraging the *tidyverse* and *slider* packages of R, we demonstrated a robust methodology for applying the KDIGO AKI criteria to real-world SCr data. This step-by-step guide, intended to be paired with the sample code, provides a foundational tool for clinical research. We hope that the readers of this paper will be motivated to conduct research on AKI more smoothly and confidently than ever.

## Supplementary Information

Below is the link to the electronic supplementary material.

Supplementary file1 (CSV 249 KB) Appendix 1. A hypothetical dataset of 1000 patients

Supplementary file2 (R 10 KB) Appendix 2. Example codes (scripts) in R (version 4.3.3)

## Data Availability

The artificially generated data used in this study are available as supplementary files. R codes (scripts) are also provided as supplementary files.
